# Development of a Short Questionnaire for the Screening for Vitamin D Deficiency in Italian Adults: The EVIDENCe-Q Project

**DOI:** 10.3390/nu14091772

**Published:** 2022-04-23

**Authors:** Rachele De Giuseppe, Chiara Elena Tomasinelli, Hellas Cena, Valentina Braschi, Francesca Giampieri, Giorgia Preatoni, Domenico Centofanti, Maria Pilar Princis, Emanuele Bartoletti, Ginevra Biino

**Affiliations:** 1 Laboratory of Dietetics and Clinical Nutrition, Department of Public Health, Experimental and Forensic Medicine, University of Pavia, 27100 Pavia, Italy; rachele.degiuseppe@unipv.it (R.D.G.); chiara.tomasinelli@unipv.it (C.E.T.); valentina.braschi01@universitadipavia.it (V.B.); giorgia.preatoni01@universitadipavia.it (G.P.); mariapilar.princis@unipv.it (M.P.P.); 2 Unit of Internal Medicine and Endocrinology, Clinical Nutrition and Dietetics Service, ICS Maugeri IRCCS, 27100 Pavia, Italy; 3 Research Group on Food, Nutritional Biochemistry and Health, Universidad Europea del Atlántico, 39011 Santander, Spain; f.giampieri@staff.univpm.it; 4 Department of Biochemistry, Faculty of Sciences, King Abdulaziz University, Jeddah P.O. Box 80200, Saudi Arabia; 5 Outpatient Service of Aesthetic Medicine, San Giovanni Calibita Ospedale Fatebenefratelli, Isola Tiberina, 00186 Roma, Italy; domenico.centofanti@tin.it (D.C.); ebartoletti@lamedicinaestetica.it (E.B.); 6 Institute of Molecular Genetics, National Research Council of Italy, 27100 Pavia, Italy; ginevra.biino@igm.cnr.it

**Keywords:** vitamin D deficiency, vitamin D screening, Nota 96 (Italian Medicines Agency), adult population, lifestyle

## Abstract

Background: To develop and validate a questionnaire for the screening of Vitamin D in Italian adults (Evaluation Vitamin D dEficieNCy Questionnaire, EVIDENCe-Q). Methods: 150 participants, attending the 11Clinical Nutrition and Dietetics Operative Unit, Internal Medicine and Endocrinology, Istituti Clinici Scientifici Maugeri IRCCS, of Pavia were enrolled. Demographic variables and serum levels of vitamin D were recorded. The EVIDENCe-Q included information regarding factors affecting the production, intake, absorption and metabolism of Vitamin D. The EVIDENCe-Q score ranged from 0 (the best status) to 36 (the worst status). Results: Participants showed an inadequate status of Vitamin D, according to the current Italian reference values. A significant difference (*p* < 0.0001) in the EVIDENCe-Q score was found among the three classes of vitamin D status (severe deficiency, deficiency and adequate), being the mean score higher in severe deficiency and lower in the adequate one. A threshold value for EVIDENCe-Q score of 23 for severe deficiency, a threshold value of 21 for deficiency and a threshold value of 20 for insufficiency were identified. According to these thresholds, the prevalence of severe deficiency, deficiency and insufficiency was 22%, 35.3% and 43.3% of the study population, respectively. Finally, participants with EVIDENCe-Q scores <20 had adequate levels of vitamin D. Conclusions: EVIDENCe-Q can be a useful and easy screening tool for clinicians in their daily practice at a reasonable cost, to identify subjects potentially at risk of vitamin D deficiency and to avoid unwarranted supplementation and/or costly blood testing.

## 1. Introduction

Vitamin D is a steroid hormone, which exerts a crucial role in the maintenance of bone and calcium homeostasis as the active vitamin metabolite, calcitriol, increases the intestinal absorption of calcium and phosphate, necessary for bone mineralisation [1,2]. About 80% of vitamin D is synthesised via sunlight exposure to ultraviolet B radiation while the remaining 20% is limited to dietary sources, including fish, dairy products, smaller amounts in eggs and meat, fortified foods, and dietary supplements [3,4]. Therefore, low outdoor physical activity and subsequent low sun exposure and poor dietary intake of vitamin D are translated into vitamin insufficiency/deficiency conditions [1]. Since vitamin D exhibits a complex multistep metabolism (hydroxylation both at the empathic and renal level) and acts through a nuclear receptor that is found in tissues not only related to calcium and bone, it has been recognised that this vitamin exerts a pleiotropic effect with many extra-skeletal targets [1,2]. Indeed, several findings [2,5] described the relationship between vitamin D intake and health outcomes such as cancer prevention and stronger immune response; diabetes or pre-eclampsia prevention during pregnancy; as well as inflammation counteraction, although not all the studies have come to the same results [2]. Thus, as reported by several findings, vitamin D deficiency is associated not only with bone health but also with an increased risk of mortality, cancer (e.g., colon, prostate, and breast cancer), cardiovascular disease, type 1 and 2 diabetes, autoimmune diseases, decreased fertility [6] and higher infection, severe COVID-19, and mortality rate [7]. Last, but not least, vitamin D deficiency was positively associated with obesity [8], especially if the fat distribution was abdominal [9]. This might be explained by the greater sedentariness of people with obesity and the lower outdoor activity, resulting in less exposure to sunlight [8]. Moreover, vitamin D is a fat-soluble vitamin and stored in the fat tissue mass [8], supporting the hypothesis that the enlarged adipose mass in subjects with obesity serves as a reservoir for vitamin D and that the increased amount of vitamin D required to saturate this depot may predispose subjects with obesity to inadequate serum 25-OH-D [10]. On the other hand, other findings reported that low levels of vitamin D could be involved as a possible mechanism in the pathogenesis of obesity [11,12,13]

Data suggest that a large part of the general population is vitamin D deficient worldwide, even in those countries where sun exposure is prolonged. Recent large observational data have suggested that about 40% of the European population is vitamin D deficient, showing serum 25-hydroxyvitamin D (25-OH-D) levels < 10 ng/mL (25 nmol/L) [14,15]. In Italy, vitamin D deficiency in the general population is very common and it is defined for serum 25-OH-D values < 20 ng/mL (50 nmol/L); however, it is recommended to maintain levels above 30 ng/mL (75 nmol/L) in risk categories [6]. Common interventions to address this deficiency include supplementation and/or fortification with either ergocalciferol (vitamin D2) or cholecalciferol (vitamin D3), but the relative efficacy of these two vitamers is unclear [16]. 

The adequacy of the prescription of vitamin D is not simple as the authorised indications foresee the administration on a daily, weekly, monthly or annually basis, up to a maximum of 600,000 IU per year [17]. Besides this, vitamin D therapy is considered a safe drug with studies showing that long-term doses of up to 10,000 IU per day are not associated with toxicity [17]. Therefore, in the recent decade, prescriptions of both assay and vitamin D supplementation have gradually increased and vitamin D3 spending has become a major concern as net spending was nearly 180 million euros in 2017, reaching first place for the International Defined Daily Dose consumption and third place overall for a pharmaceutical spending agreement [17]. Later on, in 2019, the Italian Medicines Agency (AIFA) issued “Nota 96” (“Prevention and treatment of vitamin D deficiency”) placing some restrictions on the prescriptions charged to the Italian National Health Service (NHS) for vitamin D3 in the context of prevention and treatment of vitamin D deficiency in adults (aged ≥18 years). The prescription was limited to certain targets at risk, including institutionalised persons, pregnant or lactating women, individuals with osteoporosis of any cause or osteopathy found not to be a candidate for remineralisation, regardless of the determination of 25-OH-D, as well as, after determination of 25-OH-D, all the subjects with serum levels of 25-OH-D < 20 ng/mL and symptoms attributable to hypovitaminosis (asthenia, myalgia, diffuse or localised pain, frequent unexplained falls), people diagnosed with hyperparathyroidism secondary to hypovitaminosis D and individuals with osteoporosis of any cause or osteopathy identified as a candidate for remineralising therapy for which correction of hypovitaminosis should be propaedeutic at the beginning of therapy [18].

Based on that, since vitamin D deficiency affects a high percentage of individuals during the lifespan and because some restrictions to prescribing procedures for vitamin D drugs were applied in Italy, it is necessary, for the clinical practice, to provide for the identification of a potential condition of vitamin D deficiency. The EVIDENCe-Q project (Evaluation VItamin D dEficieNCy Questionnaire) aimed at developing a questionnaire to screen the adequacy of vitamin D status in Italian adults. Precisely, this original article describes the Evaluation VItamin D dEficieNCy Questionnaire, its validation and identification of cut-offs for screening of severe deficiency (25-OH-D < 10 ng/mL), deficiency (10 ng/mL ≤ 25-OH-D < 20 ng/mL) and insufficiency (20 ng/mL ≤ 25-OH-D < 30 ng/mL) in Italian adults according to the “Italian Society for Osteoporosis, Mineral Metabolism and Bone Diseases” (SIOMMMS) guidelines on definition, prevention and treatment of inadequate vitamin D status [19]. Screening is an important part of preventive medicine and EVIDENCe-Q wishes to contribute to being a screening tool useful to identify subjects potentially at risk of vitamin D deficiency early enough to provide treatment and avoid or reduce symptoms and consequences, improving health outcomes of the population at a reasonable cost.

## 2. Materials and Methods

### 2.1. Subjects 

For this study, we approached 150 consecutive patients (112 females and 38 males), attending the Clinical Nutrition and Dietetics Operative Unit, Internal Medicine and Endocrinology, Istituti Clinici Scientifici Maugeri IRCCS, of Pavia (Italy), from March 2020 to November 2020, according to the following inclusion criteria: aged ≥18; absence of neoplasms; no bariatric surgery; able to sign the informed consent. All patients were referred to ICS Maugeri di Pavia for lifestyle interventions for Non-Communicable Diseases (NCDs) prevention (primordial and primary) and management (secondary prevention). 

During the visit, clinical history was recorded and nutritional assessment was performed, including anthropometric measurements (e.g., weight, height and waist circumference). Body Mass Index (BMI) was hereafter calculated. 

25-OH-D levels (ng/mL) were routinely measured as part of the nutritional assessment in all patients attending the Clinical Nutrition outpatients’ service. Furthermore, at the end of the visit all patients were invited to fill out the EVIDENCe-Q questionnaire using Google Forms, a web-based app used to create forms and surveys, included as part of the free Google Docs Editors suite, offered by Google [20]. All the questionnaires were then downloaded as a Microsoft Excel sheet, with the maximum guarantee of anonymity inherent in the web-survey format that prevents sensitive data from being traced [21].

The study was conducted according to the guidelines of the Helsinki Declaration and approved by the ICS Maugeri Ethics Committee of Pavia (protocol number 2385 CE). The EVIDENCe-Q project was also registered on www.clinicaltrials.gov (accessed on 1 October 2019, Identifier: NCT04404842).

### 2.2. Anthropometric Measurements

Weight and height were measured according to standard conditions. Bodyweight was measured with subjects wearing only their underwear and without shoes using a steelyard scale (precision ± 100 g); body height was measured on subjects without shoes using a stadiometer (precision ± 1 mm). BMI was then calculated as a ratio between weight and height squared with weight in kilograms and height in metres. Waist circumference (cm) was measured to the nearest centimetre with a flexible steel tape measure with participants standing, with crossed arms, placing their hands-on opposite shoulders. After gently exhaling, the abdominal waist circumference was measured on the horizontal plane between the lowest portion of the rib cage and the uppermost lateral border of the right ilium. 

### 2.3. Questionnaire Design and Scoring 

We developed the self-administered 20-item EVIDENCe-Q to screen the adequacy of vitamin D status in Italian adults. We included multiple-choice questions (13 were in dichotomous and 7 in polytomous mode) investigating factors affecting vitamin D levels production/intake, absorption and metabolism. In particular, we investigated (i) geographical information on the place of residence (north; south; central Italy and urban; peri-urban area residence); (ii) skin phototype (I–IV); (iii) regular outdoor physical activity (at least 150 min/week of moderate-intensity or at least 75 min/week of vigorous-intensity) (yes; no), according to the Italian Health Minister guidelines [22]; (iv) exposure to sunlight for at least thirty minutes, specifying how many times a week (0–7 times) and if during the 10:00 a.m.–3:00 p.m. slot (yes; no); (v) habitual use of sunscreen or cosmetics with a sun protection factor (SPF), specifying if the SPF ≥ 15 (yes; no; only during summer) and the frequency of use of sunscreen during sun exposure (one time; two times; three or more times); (vi) monthly use of UV tanning lamps (1 ≤ time; 2–3 times; 4–5 times); (vii) consumption of foods containing vitamin D (daily consumption of at least one portion of whole milk and vitamin D fortified foods; weekly consumption of at least three portions of fish; weekly consumption of at least two eggs; weekly consumption of at least two portions of dairy products); (viii) presence of certain pathologies that interfere with the production and absorption of vitamin D (e.g., liver failure, renal failure, nephrotic syndrome, hyperparathyroidism, intestinal malabsorption including Crohn’s disease, ulcerative colitis, celiac disease, cystic fibrosis, eating disorders) (yes; no); (ix) drug therapies (e.g., anticonvulsants, antipsychotics, glucocorticoids, immunosuppressive corticosteroids, anti-retroviral, weight-loss drugs, cholesterol-lowering drugs, laxatives) (yes; no); (x) use of multivitamin supplements or supplements containing vitamin D (yes; no).

We then calculated the EVIDENCe-Q score ranging from 0 (equivalent to the best vitamin D status) to 36 (equivalent to the worst vitamin D status). In particular, responses assuming behaviour that did not lead to a vitamin D deficiency were assigned a score of zero, while those that assumed behaviour potentially resulting in vitamin D deficiency were assigned a score of 1 (if the response mode was dichotomous) or greater than 1, increasing by one unit for each answer that assumed a progressively worse behaviour affecting vitamin D status. 

### 2.4. Vitamin D Levels Assessment

For each patient, we considered the serum levels of 25-OH-D (ng/mL) reported in the medical record that had been routinely determined by a one-step delayed-action immunoassay using chemiluminescent microparticles immunoassay (CMIA) technology, using the relevant commercial kits (Alinity i 25-OH vitamin D reagent kit, Abbott Laboratories Diagnostics Division, Abbott Park, IL, USA) on ARCHITECT i system because they were part of the diagnostic and therapeutic procedures of the Clinical Nutrition and Dietetics Operative Unit. According to the manufacturer, the functional sensitivity of the assay is 8.8 nmol/L (3.5 ng/mL), and the linearity through the measurement range is between 8.8 nmol/L and 385.5 nmol/L (3.5 ng/mL to 154.2 ng/mL). The limit of blank (LoB), the limit of detection (LoD) and the limit of quantitation (LoQ) are 6.0 nmol/L (2.4 ng/mL); 8.8 nmol/L (3.5 ng/mL); and 8.8 nmol/L (3.5 ng/mL), respectively.

To evaluate the vitamin D status of each patient we adopted the 25-OH-D cut-off values proposed by SIOMMS [19]. Severe deficiency was reported for 25-OH-D < 10 ng/mL levels (<25 nmol/L); deficiency for 25-OH-D between 10–20 ng/mL (25–50 nmol/L) levels; insufficiency for 25-OH-D between 20–30 ng/mL (50–75 nmol/L) levels; adequacy for 25-OH-D between 30–100 ng/mL (75–125 nmol/L) levels and toxicity for 25-OH-D > 100 ng/mL (>125 nmol/L) levels [19].

### 2.5. Statistical Analysis

Descriptive results were shown as mean value and standard deviation (SD), median value and minimum (min.) and maximum (max.) values. The chi-square test was adopted for comparing BMI status between males and females, while the *t*-test was used for the difference in mean values in men and women.

Analysis of variance (ANOVA) was used to analyse the mean value of the score in the three classes of vitamin D status (severe deficiency, deficiency and insufficiency).

To measure the degree to which the instrument is valid, the correlation coefficient between the 25-OH-D levels and the score obtained from the questionnaire was computed. Furthermore, to better describe the relationship between the two variables we fitted a quadratic regression.

To identify the threshold values useful to discriminate between subjects at risk of severe deficiency (25-OH-D < 10 ng/mL or <25 nmol/L), deficiency (25-OH-D < 20 ng/mL or <50 nmol/L) and insufficiency (25-OH-D < 30 ng/mL or <75 nmol/L) analysis of the Receiver Operating Characteristics (ROC) curve was adopted. Significance was set for *p* < 0.05. All the analyses were performed using STATA software version 16 (College Station, TX, USA).

## 3. Results

Sample characteristics, including demographic variables, BMI and vitamin D status as well as the EVIDENCe-Q score are shown in Table 1. In particular, the participants were overweight (mean ± SD BMI, 26.9 ± 5.78 Kg/m^2^), with no significant gender difference (*p* = 0.15). Subjects showed underweight (BMI < 18.5 Kg/m^2^) in 2.7% of cases (*n* = 4); normal weight (18.5 ≤ BMI < 25 Kg/m^2^) in 37.3% of cases (*n* = 56); overweight (BMI 25 ≤ BMI < 30 Kg/m^2^) in 34.0% of cases (*n* = 51) and obesity (BMI ≥ 30 Kg/m^2^) in 26% of cases (*n* = 39). Also, in this case, the frequencies of underweight, normal weight, overweight and obesity did not differ significantly between males and females. 

Mean (±SD) 25-OH-D levels of the study population (24.6 ± 10.74 ng/mL) showed that on average subjects did not show an adequate vitamin D status (cut-off: 25-OH-D ≥ 30 ng/mL or ≥75 nmol/L), according to the Italian Society for Osteoporosis, Mineral Metabolism and Bone Diseases reference values (SIOMMS) [19]. No significant gender differences were observed for vitamin D levels, as well as for the EVIDENCe-Q score (*p* = 0.48). 

EVIDENCe-Q score was then analysed to 25-OH-D levels. Analysis of variance (ANOVA) highlighted a significant difference (*p*-value ANOVA < 0.0001) in the EVIDENCe-Q score among the three classes of vitamin D status (“severe deficiency”: 25-OH-D < 10 ng/mL or <25 nmol/L; “deficiency”: 10 ≤ 25-OH-D < 30 ng/mL or 25 ≤ 25-OH-D < 75 nmol/L; “adequate”: 25-OH-D ≥ 30 ng/mL or ≥75 nmol/L, Table 2), according to the SIOMMS reference values [19]. It should be noted that, in the case of the ANOVA, the “deficiency” class included both subjects with 25-OH-D levels <20 ng/mL (<50 nmol/L) and subjects with 25-OH-D levels < 30 ng/mL (<75 nmol/L), according to the SIOMMS reference values [19]. Specifically, the mean (±SD) score was higher in the “severe deficiency” class (23.3 ± 3.99) and lower in the “adequate” one (16.2 ± 2.83), with a statistically significant decreasing trend (*p*-value trend test <0.0001). 

**Figure 1:**
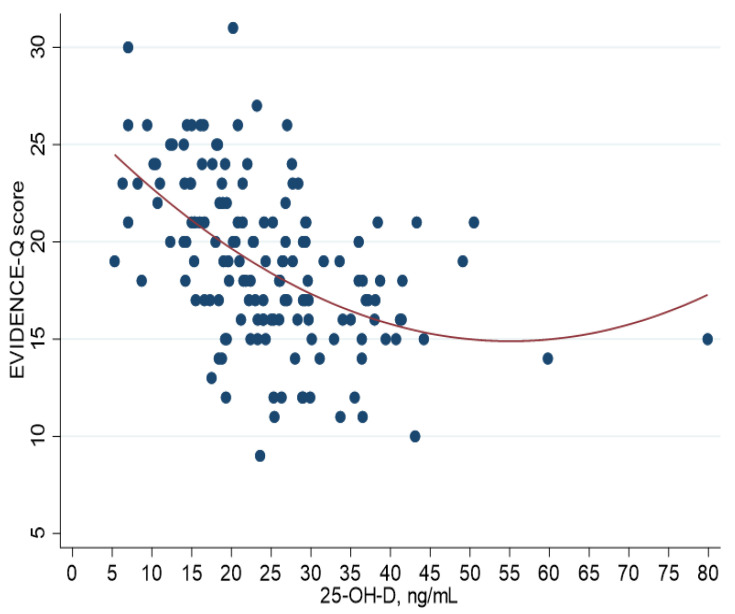
Scatterplot of EVIDENCE-Q score (*y*-axis) versus serum 25-hydroxyvitamin D (25-OH-D) levels (*x*-axis) with quadratic fitting.

Furthermore, the correlation between EVIDENCe-Q score and 25-OH-D levels was moderate, negative and significantly different from zero (r-Pearson = −0.465, *p* < 0.0001), but a quadratic regression better described the relationship between the two variables (quadratic term *p*-value = 0.002), as also shown in the scatter plot (Figure 1).

In the range of values below the acceptable threshold that describes an adequate state of vitamin D, the SIOMMS [19] has elaborated the cut-off/reference interval values useful to discriminate among subjects at risk of “severe deficiency” (25-OH-D < 10 ng/mL or <25 nmol/L), “deficiency” (10 ng/mL ≤ 25-OH-D < 20 ng/mL or 25 nmol/L ≤ 25-OH-D < 50 nmol/L) and “insufficiency” (20 ng/mL ≤ 25-OH-D < 30 ng/mL or 50 nmol/L ≤ 25-OH-D < 75 nmol/L). In this context, the analysis of the Receiver Operating Characteristics (ROC) curve was applied to investigate the optimal cut-off of the EVIDENCe-Q score that maximised the difference between the true positives (e.g., the proportion of individuals who have an altered value of the score between all those deficient) and false positives (e.g., the proportion of individuals who, despite having an altered value of the score, are not deficient) to identify the three classes of vitamin D status (“severe deficiency; “deficiency”; “insufficiency”).

Concerning the “severe deficiency” class (Table 3), the ROC curve analysis identified successfully a value of 23as the best threshold value for EVIDENCe-Q score describing subjects with 25-OH-D levels < 10 ng/mL (<25 nmol/L). According to this threshold, the prevalence of “severe deficiency” of vitamin D (EVIDENCe-Q score ≥ 23) was 22% of the study population. Similarly, a value of 21 was identified as the best threshold of the EVIDENCe-Q score describing subjects with 25-OH-D levels < 20 ng/mL (<50 nmol/L) suggestive of “deficiency” (Table 3), According to this threshold, the prevalence of “deficiency” of vitamin D (23 ≥ EVIDENCe-Q score ≥ 21) was 35.3% of the study population. A value of 20 was detected for the best threshold of the EVIDENCe-Q score that identified “insufficiency” (Table 3), including subjects with 25-OH-D levels < 30 ng/mL (<75 nmol/L). According to this threshold, the prevalence of subjects showing insufficient levels of 25-OH-D (20 ≥ EVIDENCe-Q score ≥ 20) was 43.3%. Finally, subjects with EVIDENCe-Q score < 20 had adequate 25-OH-D levels. As reported in Table 3, looking at all three curves, it is clear that the EVIDENCE–Q is fairly accurate and consistent.

Finally, Table 4 shows the comparison of the prevalence of vitamin D severe deficiency, deficiency and insufficiency according to 25-OH-D serum levels and to the EVIDENCe-Q score cut-offs identified by the ROC curve analysis. The comparison of the prevalence showed that the best fit was for “deficiency” (EVIDENCe-Q score ≥ 21 and 25-OH-D < 20 ng/mL or <50 nmol/L).

## 4. Discussion

Vitamin D is a hormone with an important role in terms of calcium and bone homeostasis as well as other several extra-skeletal effects [2,4]. The deficiency of vitamin D is very common in the Italian population [6]; prescriptions of both assay and supplementation increased more and more in the recent decade, becoming a major concern and reaching first place for consumption and second place for conventional pharmaceutical spending [17]. In 2019, AIFA issued “Note 96” which limits the prescriptions of vitamin D3 to be paid by the Italian NHS in the context of the prevention and treatment of vitamin D deficiency in the adult population [18]. Because of these restrictions, it is evident that it is necessary to identify tools that are low-cost, quick and simple to use, aimed at screening the potential vitamin D deficiency status as early as possible, guaranteeing the possibility of further and timely diagnostic and therapeutic interventions. 

In the present study, we described the EVIDENCe-Q questionnaire as a simple, easy and quick instrument for the healthcare professional who wishes to estimate vitamin D status in individuals. In particular, we developed cut-off values for the adult population of northern Italy to identify the presence of (i) severe deficiency; (ii) deficiency and (iii) insufficiency of vitamin D, according to the SIOMMS guidelines [19]. 

To date, there are several country-specific questionnaires aimed at investigating vitamin D deficiency in different and specific population groups (e.g., women, athletes, pregnancy, elderly) [23,24,25,26,27]. However, to the best of our knowledge, the EVIDENCe-Q is the first screening questionnaire simultaneously evaluating, in the general adult population, factors affecting vitamin D levels production/intake, absorption and metabolism such as (i) geographical information on the place of residence; (ii) skin phototype (I–IV); (iii) regular outdoor physical activity; (iv) exposure to sunlight; (v) habitual use of sunscreen/cosmetics with SPF; (vi) use of UV tanning lamps; (vii) consumption of foods containing vitamin D; (viii) presence of certain pathologies interfering with the production and absorption of vitamin D; (ix) drug therapies; (x) use of multivitamin supplements or vitamin D supplements. 

The questionnaire consisted of 20 items scoring from 0 (equivalent to the best vitamin D status) to 36 (equivalent to the worst vitamin D status); the score was analysed to 25-OH-D serum levels. 

The correlation analyses between EVIDENCe-Q score and 25-OH-D levels demonstrated an inverse relation (r-Pearson = −0.465, *p* < 0.0001) indicating the good behaviour of the instrument, even if a better fit was provided by a quadratic interpolation of the scatterplot, but in the same direction, with decreasing score values at increasing of vitamin D levels. The score also differed significantly (*p* < 0.0001) among subjects reporting severe deficiency (25-OH-D < 10 ng/mL), deficiency (10 ≤ 25-OH-D < 30 ng/mL) and adequate levels of vitamin D (25-OH-D ≥ 30 ng/mL).

Although the questionnaire seems to identify true negatives better than true positives as well as it seems to be most sensitive in identifying those with serum levels of 25(OH)D in the range of deficiency, the ROC curve analysis showed an EVIDENCe-Q score of 23 as the optimal cut-off value for identifying severely deficient subjects; a score of 21 identifying subjects with vitamin D deficiency, and a score of 20 describing subjects with vitamin levels D insufficiency. Furthermore, findings suggested that the best fit was for “deficiency” with an EVIDENCe-Q score ≥ 21 corresponding to 25-OH-D levels below 20 ng/mL (50 nmol/L). It is interesting to note that the cut-off value of 20 ng/mL (50 nmol/L) for 25-OH-D was also indicated by AIFA Nota 96 as the threshold for the prescription of vitamin D supplementation by the Italian NHS as it is considered the cut-off beyond which an adequate intestinal absorption of calcium and control of PTH levels are guaranteed in almost all of the population [18]. Indeed, the Institute of Medicine (IOM) Committee to Review Dietary Reference Intakes for vitamin D and Calcium indicated that 25-OH-D serum levels equal to or greater than 20 ng/mL (50 nmol/L) are sufficient to optimise the skeletal effects of vitamin D in 97.5% of the population [28]. Thus, literature data are univocal recommending vitamin D treatment in all subjects with 25-OH-D serum levels <20 ng/mL (50 nmol/L) but are controversial for values between 20 and 30 ng/mL (50–75 nmol/L) [6].

The EVIDENCe-Q questionnaire has some strengths that make it broad and easy to use in clinical practice. First of all, the questionnaire can be self-administered and has only 20 multiple-choice items, which makes it quick and easy to use. It should be noted that the questionnaire, although self-administered, is a tool of the healthcare professional, who at the time of sending it to the patient knows their weight, height and age. Therefore, the healthcare professional evaluates the result by taking into account possible confounding factors, including the BMI status. Second, EVIDENCe-Q covers young adults, adults, as well as older adults as the age of the study population ranges between 19 and 90 years. Third, the questionnaire was built on a population with a very wide range of BMI (17.5 Kg/m^2^–46.1 Kg/m^2^) since it included subjects underweight (BMI < 18.5 Kg/m^2^) overweight (BMI 25–29.9 Kg/m^2^) as well as subjects with mild and severe obesity (respectively BMI≥ 30 Kg/m^2^ and BMI ≥ 40 Kg/m^2^). Indeed, evidence shows, as reported above, that subjects with obesity have a higher prevalence of vitamin D deficiency [8]. Lastly, the EVIDENCe-Q questionnaire also investigates vitamin D supplementation prescription, considering lifestyle, dietary habits and clinical condition, to which to adapt the dosage. Therefore, the questionnaire likely represents a useful tool in clinical practice also for monitoring the prescribed therapy since the control of 25-OH-D serum levels is not recommended earlier than six months, apart from some exceptions [6]. 

The study has a few limitations, including unbalance of females and males, since about 75% of the population enrolled were women, who typically attend outpatient services for chronic diseases treatment more often than men as already described by other authors [29]. Furthermore, the study population belongs almost entirely to northern Italy. For this reason, to make the questionnaire usable throughout the national territory, the questionnaire will be validated by also including subjects from the regions of central and southern Italy. Again, we investigated the habitual use of sunscreen/cosmetics with SPF, specifying if SPF ≥ 15 and the frequency of use of sunscreen during sun exposure. However, we did not ask a specific question about whether the skin surface or parts of the body were exposed to sunlight (or UV lamps) without protective measures, representing a source of bias. Last but not least, the not fully satisfactory results of ROC analysis may be explained by the fact that this is a preliminary study and by the relatively small sample size. Therefore, there is still room for improving the questionnaire and we are planning to extend the data collection to enhance the questionnaire structure.

## 5. Conclusions

This easy-to-use questionnaire demonstrated a good relationship with the gold standard vitamin D level, showing potential for good decision support in clinical practice for screening for vitamin D deficiency in asymptomatic adults. 

Vitamin D is essential for several reasons [2]. Prevention of insufficiency (25-hydroxyvitamin D concentration ≤ 20 ng/mL) generally requires blood testing and/or supplementation, however, blood testing is costly, and unnecessary supplementation might lead to vitamin D overload with indefinite long-term consequences. The EVIDENCe-Q, which aims at identifying Italian adults at risk of vitamin D insufficiency, is useful for an easy assessment in day-to-day clinical practice, avoiding unwarranted supplementation and/or blood testing to target and treat at-risk individuals.

## Figures and Tables

**Table 1:** General characteristics of the study population. Data are described as mean value and standard deviation (SD), median value and minimum (min) and maximum (max) values. Differences between men and women were also reported.

	Total (*n* = 150)			Men (*n* = 38)			Women (*n* = 112)			
	Mean (SD)	Median	Min–Max	Mean (SD)	Median	Min–Max	Mean (SD)	Median	Min–Max	*p*
**Age** (years)	55.3 (19.5)	57.5	19–90	59.1 (19.16)	60.5	20–89	54 (19.53)	55	19–90	0.162
**Weight** (kg)	73.5 (17.59)	70	42–125.3	85 (16.62)	82.5	58–120	69.6 (16.21)	66	42–125.3	<0.01
**Height** (m)	1.7 (0.08)	1.6	1.5–1.9	1.7 (0.06)	1.8	1.6–1.9	1.6 (0.06)	1.6	1.5–1.8	<0.01
**BMI** (Kg/m^2^)	26.9 (5.78)	26.1	17.5–46.1	28.1 (5.78)	27.3	19.4–40.6	26.5 (5.75)	25.6	17.5–46.1	0.147
**WC** (cm)	91.9 (15.51)	90	61.5–134	100.4 (13.8)	101	73–125	89.1 (15.06)	89	61.5–134	<0.01
**25-OH-D** (ng/mL)	24.6 (10.74)	23.3	5.3–79.9	22.4 (8.71)	22.2	7–38.4	25.4 (11.28)	24	5.3–79.9	0.129
**EVIDENCE-Q score**	18.9 (4.21)	19	9–31	18.5 (4.99)	18.5	9–31	19.1 (3.93)	19	10–27	0.479

Legend. BMI: body mass index; WC: waist circumference. Significance *p* < 0.05 (Males vs. Females), *t*-test for the difference of mean values in men and women.

**Table 2:** EVIDENCE-Q score by vitamin D status, according to the SIOMMS reference values [19]. Data are described as mean value and standard deviation (SD), median value and minimum (min) and maximum (max) values.

EVIDENCE-Q Score	Mean (SD)	Median	Min–Max	*p*-Value
Severe deficiency (25-OH-D < 10 ng/mL)	23.3 (3.99)	23	18–30	0.0001
Deficiency (10 ≤ 25-OH-D < 30 ng/mL)	19.4 (4.14)	20	9–31	
Adequate (25-OH-D ≥ 30 ng/mL)	16.2 (2.83)	16	10–21	

Legend. Significance *p* < 0.05, Analysis of Variance (ANOVA).

**Table 3:** Summary of ROC curve analysis results using the three reference variables.

	Severe Deficiency (25-OH-D < 10 ng/mL)	Deficiency (25-OH-D < 20 ng/mL)	Insufficiency (25-OH-D < 30 ng/mL)
**Optimal operating slope**	1	1	1
**Optimal cut-off**	23	21	20
**Optimal sensitivity**	0.625	0.6182	0.5214
**Optimal specificity**	0.8028	0.8	0.8788
**Clinical information statistic**	0.4278	0.4182	0.4002
**Area under the ROC Curve**	0.8129	0.7702	0.7682
**SE of Area (Hanley)**	0.0697	0.0423	0.0418
**Sample size**	150	150	150

**Table 4:** Prevalence comparison.

	(25-OH-D)	EVIDENCE-Q Score Cut-Off
	*n* (%)	*n* (%)
**Severe deficiency < 10 ng/mL**	8 (5.3)	33 (22)
**Deficiency < 20 ng/mL ^a^**	55 (36.7)	53 (35.3)
**Insufficiency < 30 ng/mL**	117 (78)	65 (43.3)

Legend. ^a^ the best fit reported by the prevalence comparison.

## Data Availability

All data presented in this study, not yet publicly archived, shall be made available through the corresponding author on request.
